# Something Old, Something New: The Modernization of the Older Americans Act

**DOI:** 10.1093/geroni/igaa057.2516

**Published:** 2020-12-16

**Authors:** Lauren Bangerter, Beth Prusaczyk, Brian Kaskie

## Abstract

The Older Americans Act (OAA) is the foremost federal law focused on the wellbeing of aging adults in the US. Since its conception 1965, the OAA has sought to optimize the lives of aging Americans, with emphasis on low-income adults, through programs that promote nutrition, transportation, support caregivers, offer employment, and combat elder abuse. This symposium will explore the modernization of the 2020 OAA, which was last reauthorized in 2016. Presentations 1 and 2 will focus on important updates to the definitions used throughout OAA (Title I). Presentation 3 will cover several noteworthy changes to improving grants for states and community programs on aging’s (Title II). Presentation 4 will provide additional context to amendments made to modernize activities for health, independence, and longevity (Title III) prioritize senior Community Service Employment Programs (Title IV) and enhance grants for Native Americans (Title V). Presentation 5 explores the modernizing allotments for vulnerable elder rights protection activities and other programs (Title IV) included changes in funding and home and community-based best practices and elder justice activities. Collectively, these presentations will provide an overview of the key changes in the reauthorization of the OAA. This work will allow GSA attendees to understand the specific efforts to modernization this critical legislation to better serve the aging US population.

